# Who Are the Superfoodies? New Healthy Luxury Food Products and Social Media Marketing Potential in Germany

**DOI:** 10.3390/foods10122907

**Published:** 2021-11-24

**Authors:** Christoph Frank Wiedenroth, Verena Otter

**Affiliations:** 1 Department of Agricultural Economics and Rural Development, University of Göttingen, 37073 Göttingen, Germany; 2 Business Management & Organisation Group, Wageningen University, Hollandseweg 1, 6706 KN Wageningen, The Netherlands; verena.otter@wur.nl

**Keywords:** new healthy luxury food products, superfoods, food quality, foodies, social media marketing, exploratory factor analysis and hierarchical cluster analysis

## Abstract

Superfoods, former traditional foods that in some cases are now regarded as new healthy luxury food products (NHLFP), have been growing in popularity in high- and middle-income societies. Despite a growing interest in superfoods, a precise definition of NHLFP, which appears to mark a subcategory of superfoods, together with a comprehensive analysis of NHLFP consumer segments does not yet exist. This is of particular relevance to managers as profound knowledge of different consumer groups is a prerequisite for the use of marketing approaches such as social media marketing. Therefore, this research proposes and validates an NHLFP definition and investigates whether promising NHLFP consumer groups can be identified based on selected psychographic and sociodemographic consumer characteristics and whether these groups are also accessible through social media marketing. A data set of 697 fruit consumers in Germany was retrieved in the time period of May to June 2020 and analyzed through exploratory factor analysis and hierarchical cluster analysis. Eleven factors and four consumer groups were identified, two of which represented favorable superfood consumer groups—one group consumed for intrinsic, health-related reasons rather than for luxury-driven motives, while the other showed tendencies to purchase superfoods for luxury reasons, thus emerging as a promising NHLFP target group. This group is relatively younger, well-educated, and highly receptive of online marketing.

## 1. Introduction

A new food product category centered on traditional food products that are “rich in compounds (such as fiber, antioxidants, or fatty acids) considered beneficial to a person’s health” [1] has recently emerged. These foods are generally described as “superfoods”, and they have become particularly popular in developed countries [2,3]. To many consumers, superfoods represent a luxury food product, and research has linked traditional luxury food products to above average product prices that function as a search attribute in comparison to other fruits and vegetables [4]. Additionally, credence attributes, such as sustainable production practices, and experience criteria, such as taste (oftentimes termed as indulgence), have already been associated with a greater luxury perception of traditional luxury food products [5,6]. This is expected to also hold true in the case of superfoods, as their taste [7] and credence dimensions [3,8,9,10] have already been proven to be important determinants for influencing consumer purchasing decisions. Yet, consumers are faced with the non-visibility of credence attributes, which at first seems to interfere with their ability to use these attributes as a luxury dimension. To overcome this, consumers use specific search and experience attributes of superfoods that are closely related to their credence-based health luxury dimensions. This, for example, led to a sharp increase in demand for purple- and blue-colored superfoods as many consumers perceive these colors to be a good health indicator and, thus, a way to visualize superfood health benefits [11]. Furthermore, consumer health-related activities since at least the beginning of this century have been linked with their desire to display a social class distinction through their food purchases and consumption [12,13] and to communicate their healthy behavior to others. Such communication increasingly takes place via social media platforms (SMPs), particularly picture-based platforms such as Instagram and Pinterest, on which the sharing of colorful and food-related content has become a common phenomenon and which have been hotbeds of many lifestyle-related food trends in the past [11,14,15].

The intention to display social class differences drives the demand for superfoods and motivates the use of the described luxury dimensions [16]. The ongoing SARS-CoV-2 (coronavirus) pandemic is likely to accelerate this trend as health awareness and time spent on meal preparation have increased in high-income countries [17,18]. In the long run, these unique luxury dimensions of superfoods are expected to become even more relevant as traditional luxury products, such as brand clothing and digital hardware, become more accessible to a wider range of social classes, and consumers who are in search of new ways to establish social distinction increasingly use the consumption of superfoods as a replacement [16]. This further emphasizes the luxury dimension of superfoods as a continuing shift that other luxury food products are unlikely to follow as they oftentimes lack the ability to visualize credence attributes. While the validity of the term “superfoods” is still contested, and systematic research on such products is limited [4,7], we rely on the findings of Franco Lucas et al. [7], Butterworth et al. [4], Groeniger et al. [16], Hartmann et al. [5,19], and others to put forth the proposition that superfoods can be considered a type of “new healthy luxury food product” (NHLFP) based on the following definition proposed for NHLFP: New healthy luxury food products (NHLFPs) are traditional food products beneficial to a person’s health that are utilized as luxury products due to their above average product price and coexistence of health-related search and experience attributes.

**Figure 1:**
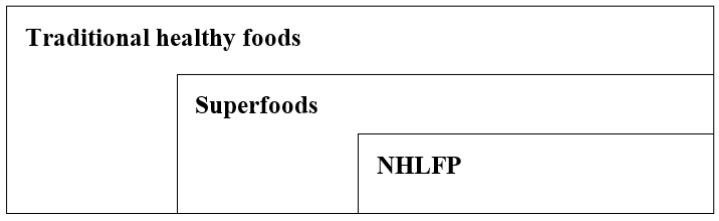
Categorization of NHLFPs.

NHLFPs also need to be understood as a product group within the greater superfood category, which itself describes a subcategory of traditional healthy foods (see Figure 1).

Effective and well-tailored marketing strategies are essential for the creation of luxury product perceptions among consumers. Such strategies have to go beyond purely emphasizing product quality characteristics to target product lifestyle dimensions that suggest unique consumption experiences [20]. Therefore, social media marketing (SMM) strategies are becoming a central tool for developing opinions as well as influencing behaviors, for example, by creating strong brand loyalty among users [21,22]. This happens because social media platforms offer a unique selling point, allowing users to create content, compare activities with peers, self-present, and distinguish themselves through these actions—in short, to make greater use of the lifestyle dimension of luxury products [23]. For non-food-related luxury product categories, such as clothing and cars, a shift toward SMM in general and influencer marketing in particular has been well established [21,24]. An even stronger development is expected to take place in the marketing of NHLFPs for two reasons. First, SMPs already influence consumer food quality perceptions, such as consumer views on different credence food attributes [25,26]. Second, superfoods have already been part of many consumer trends that have developed across SMPs in the past [11,14,15] and further research supports the importance of SMPs for future superfood marketing [27]. As NHLFPs might represent a subcategory of superfoods, we expect similar dynamics to be present there. As these dynamics develop, the affected marketers will need a well-grounded understanding of exactly what constitutes NHLFPs and what NHLFP consumer segments might look like in order to design suitable SMM-based strategies. However, to the best of our knowledge, scientific analysis has not yet provided this level of detail. On the one hand, only a few studies have identified superfood consumer groups, and of this research, even fewer have linked observable consumer characteristics to possible SMM strategies [5,7,19,28]. On the other hand, research on traditional luxury food products contains conflicting evidence; as Hartmann et al. [5] concluded, “buyers of organic and fair trade food products have to be differentiated from buyers of expensive premium food brands.” Translated into the context of NHFLPs, this implies that the consumers of superfoods who are more likely to ascribe higher importance to food attributes such as organic and fair production practices [7,29] are unlikely to be the same people who consume NHFLPs for luxury reasons [5]. This observation is strengthened because superfood consumer segments are described as highly price sensitive [29], thereby contradicting the presumed NHLFP consumption motives. Additionally, the desire to display social distinction, which we outlined as an important factor for NHLFP demand and, therefore, marketing strategies, has not thus far been incorporated into any research. These shortcomings are troubling as SMM, which is targeted at possible superfood and NHLFP consumer segments, needs to be well tailored because different social groups differ in how they share food-related information [29,30].

Against this background, we raise the following research questions: Do empirics support the proposed definition of NHLFP based on the existing unsystematic knowledge? Is there a specific consumer group for new healthy luxury food products that can be defined by consumer psychographics? Is such a group particularly accessible for SMM? For this purpose, survey data from 697 consumers in Germany was collected to research blueberries as an NHLFP case. Data analysis employed exploratory factor analysis and hierarchical cluster analysis. The empirical results offer insights on promising consumer segments for future marketing strategies. This is of interest to managers and scientists alike who are involved in the online marketing of luxury food products or who want to learn more about the future direction of online food marketing.

## 2. Case and Conceptual Background

### 2.1. Superfoods—The Case of Blueberries in Germany

There is no common understanding among consumers as to the variety of products that belong to the category of superfoods [16]. Yet, blueberries have been at the forefront of the developing superfood trend from the very beginning. They belong to the most often mentioned superfoods and strongly reflect the health benefits that are associated with superfoods [4,31,32,33]. At the same time, blueberries incorporate the necessary search- and credence-related luxury dimensions in line with our proposed definition of NHLFPs. Blueberries are perceived as a form of luxury product and consumers regard them as above average in expense [4]. This emphasizes the search attributes of blueberries—in this case, the product price—that impact consumer food choices. Furthermore, the health benefits of blueberries and the presence of organic labeling are found to influence consumer purchasing behavior. This highlights consumer awareness of credence attributes prior to making a purchase decision [34]. Blueberries are also highly present across social media platforms, and multiple health-related movements have developed on the platforms in the past, such as the recent “standout color” trend in which pictures of purple and blue foods were widely shared across social media platforms [11,35]. Germany makes a well-suited case study as residents are very familiar with superfoods and the demand for blueberries is high but still rapidly growing. Germany belongs to the group of high-income countries among which we expect the superfood trend to be most distinct [36,37]. Furthermore, within Germany, blueberries are advertised considerably more often than are other superfoods, such as avocados and kakis [38].

### 2.2. Exploratory Research Concept

We draw on insights from different scientific research fields involved with consumer research to provide a reason for this exploratory research design, namely that of luxury food products and superfood consumption patterns as well as general online marketing [5,7,11,39].

Research on traditional luxury food products indicates that corresponding consumer segments attribute high importance to the prestige and social status that comes with the consumption of a certain luxury food product [5,40]. As NHLFPs incorporate traditional luxury product dimensions, these characteristics are likely to mark an important determinant of the corresponding consumer segment. Thus, consumer inclinations to acquire higher social status through their own food consumption will be included in a later exploratory research design. Furthermore, if consumers want to utilize blueberries as a luxury food, they need to be aware of corresponding product attributes, such as extrinsic (e.g., product price) and intrinsic (e.g., content of nutritive substances) blueberry characteristics, and perceive these attributes as luxury dimensions [5,19]. Yet, by themselves, traditional luxury food consumer segments are unlikely to make an ideal target group. They tend to discard credence attributes as a luxury dimension [5], but we previously identified these attributes as important to NHLFP sales. This requires an investigation of a second research string that analyzes food consumer segments outside of the luxury food product category. As NHLFPs are assumed to represent a section of the greater category of superfoods, incorporating superfood consumer characteristics promises to be a good way forward. A first distinction must be made between different superfood consumer “personalities” that consumers choose in order to showcase self-identity, oftentimes tied to a larger group identity [28,41]. These personalities, which are all highly involved with food topics, are differentiated by Sikka [28] into “consumers” who are primarily interested in nutritional benefits and “subcultures” or “lifestyles” who consume superfoods to display self-identity [28,42,43]. In particular, subcultures exhibit higher social entry barriers in which consumption functions as a means of expressing social belonging, at times articulated through Internet-based networks [28]. As described by Sikka [28], these subcultures seem to fit the “foodie” consumer segment, a term that describes consumers who are highly interested in and passionate about food and who like to share eating habits with their social networks [29,44]. Foodies display above average receptiveness to food credence attributes as they are exceedingly health conscious and sensitive toward environmentally sustainable production practices [45]. They favor preparing and eating food with friends, and they share their eating experiences on social media more often than do other consumer groups. This fits the consumption pattern of superfoods as well as our hypothesized consumption motives for NHLFPs [29,33,44,45]. Defining the characteristics of this consumer segment, such as their health and environmental awareness, food knowledge, and tendency to compare eating habits with friends, should be incorporated into a later exploratory research design. Foodies are also social media adept and display a certain affinity toward online marketing content [29]. In line with the outlined NHLFP consumption and marketing dynamics, this justifies the inclusion of both characteristics. However, some limitations apply to foodies as an ideal NHLFP target group. First, foodies are found to be highly price sensitive, which runs counterfactual to our expected NHLFP consumption patterns [29]. Second, other than their affinity for SMPs, we know very little about how foodies perceive media content and the presented advertisement sources. As SMM marks an important future marketing determinant, knowledge of NHLFP consumer perceptions of media sources becomes an important factor for success.

For that reason, we borrow from a third and final string of research that busies itself with successful forms of online marketing. The ultimate success of online marketing is dependent on consumer trust toward a given media. Consumers pursue alternative media sources, such as online information channels, oftentimes today called online influencers, if they tend to distrust traditional media sources [46,47]. Thus, high levels of distrust within a given media channel cast doubt on the success of a particular marketing strategy and indicate the direction consumers are traveling toward in the use of new channels, which should, therefore, be included in a later research design.

## 3. Material and Methods

### 3.1. Study Design

Based on our argument of what a possible NHLFP consumer segment could look like, data were collected from German fresh fruit consumers. The final questionnaire was comprised five different subsections and was based on a pretest with 114 German fruit consumers conducted in January 2020. At the beginning of the questionnaire, information was briefly provided on the content of the questionnaire. As blueberries are known by many different names in Germany, clarification was offered. Next, questions related to quota requirements were answered, followed by the first subsection on fresh fruits. In line with general food consumption literature, this subsection contained queries on respondent’s environmental awareness (partly based on [48,49]), attitude toward food, fruit consumption motives, fruit involvement, consumption uncertainty, and situational fresh fruit-related factors. The following subsection, which was also rooted in the literature on traditional luxury food products and addressed blueberries in particular, included consecutive questions on intrinsic and extrinsic blueberry product characteristics, experience and quality characteristics, and situational blueberry-related factors. The third section addressed respondents’ health awareness (based on [50]), while the fourth section queried media constructs based on the online marketing literature in order to prevent response bias. In this section, frequency of Internet usage had to be answered, followed by questions on respondent’s Internet marketing affinity, social media involvement (based on [51]), and trust in and use of different media sources (based on [46,47]). Last, section five retrieved sociodemographic characteristics. Questions concerning fresh fruit and blueberry characteristics as well as media affinity were measured using five-point Likert scales stretching from “fully agree” (+2) to “fully disagree” (−2), while sociodemographics were queried through nominally and metrically scaled questions. Final data collection took place between May and June 2020 with the assistance of an online panel provider. To prevent contributors from participating multiple times, online survey links could only be used once. Respondents younger than 18 years old were excluded for ethical reasons. Respondents who consumed fresh fruit less often than once a week were also excluded to ensure that participants were sufficiently familiar with fruit-related content. Additional quotas were established for gender and age to guarantee representativeness of the overall German population [52]. In total, 763 respondents completed the questionnaire, of which 65 were excluded for failing to provide the correct answer to the quality control question (The question was stated as follows: “This is a quality assurance for our questionnaire. Please click the option “fully agree” only). If respondents greatly exceeded or fell far below the mean processing time period of 20 min and 35 s to complete the questionnaire, their responses were also excluded from the final data sample. In total, 697 observations remained in the cleaned data set.

### 3.2. Statistical Analysis

After descriptive analyses, an exploratory factor analysis was conducted for dimensional reduction. This is necessary as each of the characteristics hypothesized to drive superfood consumption behavior in Section 2.2 requires measurement using multiple items, which factor analysis reduces into a smaller and, thus, better interpretable number of factors [53]. This exploratory factor analysis assumed the presence of correlation between different factors, for which reason the oblique rotation method (Promax) and Kaiser Normalization were used [53]. Using principal component analysis, all factors scoring above 1 were included. The identified items were then tested regarding their fit for factor analysis by employing the Bartlett test for sphericity to test for the assumed null hypothesis of no correlation between the chosen items [53]. Additionally, the Kaiser–Meyer–Olkin (KMO) criteria, also described as a measure of sampling adequacy (MSA), were utilized considering the lowest suggested value of 0.6 [53]. 

Based on the previously identified factors and given the relative small sample size [54,55], a hierarchical cluster analysis was performed. As cluster analysis identifies specific homogeneous consumer groups within heterogeneous populations [53], it is an appropriate method for the setting of this research, which seeks to identify NHLFP consumer groups through shared psychographic characteristics and SMM potential. Furthermore, cluster analysis is well established in consumer behavior and marketing research, which allows for comparison of results across studies. The single-linkage method was first applied in order to discharge outliers, which was then followed by the WARD method to determine the ideal quantity of clusters. The results obtained were reconfirmed by interpreting the dendrogram as well as the scree plot outputs. After this, and following the recommendations of Backhaus et al. [53], a k-means cluster analysis was performed to reconfirm previously made observations, while a discriminant analysis indicating the clusters’ Eigenvalues, Wilk’s lambda value, and corresponding significant levels as well as Chi-Quadrat values contributed additional assurance. Thereafter, different multiple group comparison tests were carried out in order to observe significant differences between clusters. First, a univariate ANOVA was employed and was added by cross tables and a post hoc multiple comparison test, in this case, the TamhaneT2 test. Last, Bonferroni corrections were used to prevent type I errors occurring from wrongly rejecting the null hypothesis. All statistical analyses were conducted using Windows IBM SPSS Statistics 26. 

## 4. Results

### 4.1. Sample Description

The descriptive statistics reveal that the final data set consisting of 697 complete observations can be considered representative of the German population with respect to gender (51% of respondents are female) and most age groups (excluding participants older than 60 years; see Table 1). The geographic places in which people live, their health consciousness (roughly 19% smoke regularly), and their average time spent on the Internet each day (191 min) are representative of the greater German population. The share of vegetarian and vegan consumers is not entirely representative with an aggregated share of 11.6%, as is the case with different income groups. Moreover, 36% of respondents have achieved an average education and 45% have a higher education degree. Both of these groups are therefore overrepresented within this data sample by 5% and 12%, respectively, compared to the German population. The respondents’ fruit consumption frequency is slightly overrepresented within this data sample, which was expected as we imposed quota requirements of consuming fruits at least once per week. Overall, this sample must be considered rather small for the later cluster analysis (see Section 4.3) [54], but still provides an interesting starting point when analyzing NHLFP consumer segments.

### 4.2. Factor Analysis

The exploratory factor analysis included all questions on the questionnaire described in Section 4.2. Sample description, which were measured through five-point Likert scales, namely the participants’ fruit attitudes and blueberry perception as well as their health awareness and media involvement. This analysis yielded a total of 11 different factors based on 50 different items (see Table 2). Cronbach’s alpha values of factors 1, 2, 3, 4, and 5 placed above 0.85, while factors 6, 7, 8, and 9 were larger than 0.65. These were then followed by factors 10 and 11, which scored lower but still within an adequate range [66]. The results of the principal component analysis indicated that a total of 65.64% of the observed variance can be explained through the derived 11-factor solution. The null hypothesis of the Bartlett test for sphericity at 1% level was rejected and correlation between the derived items was assumed to be present. The results of this exploratory factor analysis were further strengthened as the KMO totals were at 0.880, which is described as meritorious by Backhaus et al. [53].

The first factor, “Media Quality Perception I”, consisted of six items in total, two of which (items 1.1 and 1.2) assessed the respondents’ journalistic quality perceptions, and two items each investigated the assumed selectivity of facts (items 1.3 and 1.4) and the selectivity of topics (items 1.5 and 1.6) by the German media when covering food-related themes. The factor “Environmental Awareness” was used to represent consumer preferences toward eco-friendly living practices (e.g., items 2.4 and 2.6) and purchasing behaviors (e.g., items 2.2 and 2.3) through seven different items in total. Third, consumers’ “Health Awareness” was displayed through six different items in which all items embodied levels of consumer health consciousness. Here, items 3.1 and 3.3 represented respondents’ general involvement with their health while items 3.2 and items 3.4 to 3.6 provided a more detailed examination of the respondents’ consciousness toward changes in their health. Factor 4, “Social Media Involvement”, was represented by five different items. These reflected consumer use of social media platforms for acquiring information on social events (items 4.1 to 4.3) or other topics that are of interest to them, such as information on companies (item 4.3) or certain products (item 4.5). Furthermore, this factor entailed consumer use of social platforms as a vehicle for social interactions (item 4.4). Factor 5 functioned similarly to factor one as an indicator of media quality perception. Yet, it provided a more nuanced view on consumer opinions on the selectivity of the German media when reporting on food topics, as three out of the four items that made up this factor reflected perceived news selectivity by the media (items 5.1 to 5.3). Next, five items made up factor 6, “Extrinsic Blueberry Characteristics”, and described consumer perceptions of extrinsic blueberry features. On the one hand, this included consumer views on the cultivation process of blueberries (items 6.1, 6.2, 6.3) and, on the other hand, reflected their perceptions of blueberry packaging designs and labels (items 6.4., 6.5). Factor 7, “Online Marketing Affinity”, centered on three different items that showed consumer familiarity with food advertising while using the Internet (item 7.3) and their likeliness to thereafter purchase the promoted products (items 7.1 and 7.2). Factor 8, “Social Comparison of Fruit Consumption”, was described by four different items that depicted consumer intention to receive social recognition from their social network when consuming healthy products (items 8.1 and 8.2), such as friends (item 8.3) and family members (item 8.4). Factor 9, “Intrinsic Blueberry Characteristics”, was represented by four different items. This factor described consumer awareness of blueberry quality parameters, such as their health benefits (item 9.2), product appearance (item 9.3), and sensory parameters. Sensory parameters were split into two sensory experiences, those of taste (item 9.1) and smell (item 9.4). Additionally, factor 10, “Luxury Perception of Blueberries”, was represented by three items that measured respondent perceptions of blueberries as a luxury good compared to other fresh fruits. For example, item 10.2 queried if consumers considered blueberries as a particularly exclusive food product, while items 10.3 and 10.1 reflected consumers’ price and social perceptions of blueberries as possible luxury dimensions. Last, factor 11, “Fruit Knowledge”, represented consumers’ self-reported fruit-related knowledge through three different items. These items represented consumer awareness of the diversity of available fruit products (item 11.1) as well as their knowledge of fruits (item 11.2, 11.3).

### 4.3. Cluster Analysis

The hierarchical cluster analysis was applied using the 11 factors as cluster-forming variables. Table 3 present the results of the cluster analysis, while Table 4 and Table 5 show the sociodemographic descriptions of the clusters. The results yielded an accurate grouping of 95.8% of the initial cases, which resulted in four different consumer clusters. Corresponding quality criteria were desirably high as Wilk’s lambda results were highly significant (*p* < 0.01), and two Eigenvalues were above 1, thus sufficiently high, while one Eigenvalue was above 0.7. By applying the Tamhane T2 post hoc multiple comparison test, significant variances among clusters across different factors were observed. (Items described in the following are mentioned in Table 2, while aggregated results are presented in Table 3).

The first cluster, Cluster A, is called the “always skeptical” (*n* = 205, average age ≈ 47 years) because affiliated consumers showed a negative inclination toward all previously identified factors. “Environmental Awareness” (μ = −0.78), “Health Awareness” (μ = −0.49), and “Fruit Knowledge” (μ = −0.83) stand out among these factors by revealing a particularly negative peculiarity. In line with the latter, levels of knowledge on intrinsic (factor 9, μ = −0.64) and extrinsic (factor 6, μ = −0.44) blueberry characteristics are the lowest compared to other clusters. Furthermore, this cluster contains the highest share of meat eaters (85.9%) and significantly the lowest share of respondents who exercise regularly (47.3%; *p* < 0.01). It also describes the only identified consumer group that is significantly male led (share of females ≈ 42.9%) in which the place of living (28.3% reside in the East of Germany) marks a noticeable difference. Last, little involvement with social media platforms (factor 4, μ = −0.12) and “Online Marketing Affinity” (μ = −0.02) are observed, while members also read newspapers and professional journals significantly less often than those in the other clusters (48.8%; *p* < 0.01).

Second comes Cluster B, the “media-skeptical light foodies” (*n* = 172, average age ≈ 48 years, share of females ≈ 56.4%). This cluster is characterized by high levels of health awareness (factor 3, μ = 0.22), with members who are characterized by a significantly higher proportion of housewives/men (8.7%; *p* < 0.01) and who exercise more often (69.8%; *p* < 0.01). Moreover, a larger proportion of members maintains a meat-extensive (72.1%; *p* < 0.1) dietary composition. In addition, cluster members show the highest level of “Fruit Knowledge” (μ = 0.39) and high familiarity with “Intrinsic Blueberry Characteristics” (μ = 0.21) but no profound knowledge of extrinsic blueberry characteristics (factor 6, μ = −0.06). High levels of “Environmental Awareness” (μ = 0.49) are also present, with a product’s degree of environmental pollution (item 2.1., μ = 1.20) and unnecessary packaging (item 2.2, μ = 1.22) especially important to members. At the same time, media reporting on food topics is viewed as insufficient (Media Quality Perception I and II; μ = −0.75, −0.96) and members less regularly use traditional information sources such as advertising brochures (59.9%; *p* < 0.01). Despite their low appreciation of news media quality in reporting on food topics, no enhanced use of alternative media sources was observed. “Social Media Involvement” (μ = 0.00) is small at best and only used for connecting with friends (factor 4, item 4.4, μ = 0.21). Likewise, members reveal low levels of “Online Marketing Affinity” (μ = −0.37), as exemplified by item 7.2: “I regularly click on advertisements that are displayed to me on the Internet” (μ = −1.61). Last, Cluster B has the highest level of household income and acknowledges blueberries to be high priced (item 10.3, μ = 0.67) and exclusive (item 10.2., μ = 0.30) while simultaneously strongly rejecting the “Luxury Perception of Blueberries” (μ = −0.12).

Cluster C, the “traditionalists” (*n* = 206, average age ≈ 57 years, share of females ≈ 53.4%), contains the largest share of people in retirement (≈40%) and has the highest average age. Clusters members use social media platforms to a significantly lower degree (*p* < 0.01) than other clusters while exhibiting the highest degree of food-related “Media Quality Perception (I, μ = 0.59 and II, μ = 0.82).” Among cluster members, daily and weekly newspapers are most frequently used (70.4%; *p* < 0.01). Yet, compared to Cluster B, which shows low media quality perception accompanied by high levels of fruit knowledge, Cluster C has among the highest levels of “Fruit Knowledge” (μ = 0.38) and knowledge on “Intrinsic Blueberry Characteristics” (μ = 0.25). This cluster is also characterized by a significantly higher familiarity with blueberries (47.5%; *p* < 0.05) and a higher share of consumers with vegetarian eating habits (12.6%; *p* < 0.1) as well as a significantly lower share of people with vegan eating habits (0.5%; *p* < 0.1). Although Cluster C has similar income and education levels compared to Cluster A, it displays no clear inclination toward “Environmental Awareness” (μ = 0.05) and “Health Awareness” (μ = −0.12). Last, members strongly negate the possibility to consume fruits for reasons of social comparison (factor 8, μ = −0.43) and consume fruits across the clusters the least often to express health awareness to friends (item 8.3, μ = −1.74). This observation is strengthened as no “Luxury Perception of Blueberries” (μ = −0.17) is observed.

Cluster D is called the “trustful luxury-seeking social foodies” (*n* = 114, average age ≈ 43 years, share of females ≈ 54.4%). The cluster-forming variables “Environmental Awareness” (μ = 0.57) and “Health Awareness” (μ = 0.78) score highest within this cluster group. Despite displaying the largest share of smokers, members exercise significantly more regularly (70.2%; *p* < 0.05) and include the second-lowest share of meat consumers. They also represent the smallest, youngest, and most student-led (17.5%; *p* < 0.1) respondent group in which every second member has achieved a high level of education. Furthermore, high “Involvement with Social Media” (μ = 0.92) and “Online Marketing Affinity” (μ = 1.27) are present. Although their possession of digital hardware and intensity of Internet usage do not stand out in comparison to the other clusters, a wide range of different social media platforms are significantly more often utilized. This includes the use of relationship networks (75.4%; *p* < 0.01), platforms for sharing pictures (51.8%; *p* < 0.01) and videos (71.1%; *p* < 0.05), blogs (36.8%; *p* < 0.01), bookmarking sides (32.5%; *p* < 0.01), and interest-based networks (19.3%; *p* < 0.01). This also fits with the high “Involvement with Social Media” (μ = 0.92) and “Online Marketing Affinity” (μ = 1.27) present within this cluster. Social media platforms are used more often for connecting with friends (factor four, item 4.4, μ = 1.10) and obtaining interest-specific information as exemplified by factor four, item 4.5: “I often use social media to inform myself about products that interest me” (μ = 0.56). For example, this can include food-related content, as members of this cluster rely on SMPs more regularly than other clusters for obtaining price and health-related information about blueberries. Moreover, factor 8, “Social Comparison of Fruit Consumption” (μ = 1.29), shows that fruits are frequently consumed in the presence of friends (item 8.2, μ = 0.48) and are used to display health-conscious behavior (item 8.4, μ = 0.10). Last, this group is the most familiar with blueberries (significant level of *p* < 0.01) and displays the strongest “Luxury Perception of Blueberries” (μ = 0.57) in which items 10.2, “Compared to other fresh fruit, blueberries are particularly exclusive” (μ = 0.89), and item 10.3 “[…] are high price […]” (μ = 0.88) receive the highest scores across clusters.

## 5. Discussion

This research set out to establish a first definition of NHFLP while identifying consumer segments which consume superfoods for luxury health reasons. Whether an NHLFP consumer segment receptive to SMM strategies could be identified was also of interest. To answer this, a data set of 697 respondents was collected, yielding eleven different factors. These factors served as a basis for hierarchical cluster analysis which resulted in four different consumer segments. 

Among the 11 factors identified, three factors—“media quality perception I”, “environmental awareness”, and “health awareness”—stood out as they particularly shaped the characteristics of the subsequent consumer groups. The latter two factors were measured through well-established scales, even though they have been discussed with little objection in the available literature [48,67,68,69]. The measurement scale of the former factor, “media quality perception I”, has also received much attention but remains subject to an ongoing discussion [46], which will deepen in the following section. In line with Prochazka and Schweiger [46], Fletcher and Park [70], and Kohring and Matthes [47], we argued in the beginning of this paper that low levels of trust in traditional media sources will lead to the use of alternative nonmainstream media sources, such as social media platforms and blogs. Yet, compared to this research, the relationship between media trust and media usage is less visible in our case. For example, there is no clear difference with respect to the use of alternative media sources between Clusters A and B, which place low levels of trust in traditional media sources, and Clusters C and D, which place high levels of trust in the same media sources. Rather, in the case of Cluster D, the link between media trust and usage runs the opposite direction as high levels of media trust and high utilization of alternative media sources, such as blogs, can be observed (see Table 4 and Table 5). Therefore, the former subdivision of media sources into traditional and nontraditional (alternative) media types in which the use of the latter is a result of a lack of trust in traditional media sources does not apply here. We assume that this is also the case for the motives of media use outside of NHLFPs or superfood consumer segments because most of the formerly traditional media sources already possess an online presence as they seek to diversify their media channel portfolios [71]. People might simply decide to use nontraditional media channels out of convenience or to think of online media channels only when the questionnaire asked about their level of trust. Second, the ways in which people stay informed is changing. Most people in high-income countries acquire information across multiple platforms at the same time, platforms in which short video clips, podcasts, and topic-specific blogs are becoming increasingly popular [57]. Therefore, it no longer seems helpful when analyzing user dynamics to label the utilization of nontraditional media channels as resulting from trust deficits toward traditional media channels. Although these dynamics are present, as evidenced by Prochazka and Schweiger [46] and Kohring and Matthes [47], they are unlikely to apply to entire media channels, such as blogs or bookmarking sides. Rather, these dynamics will hold true for subsections within the greater group of nontraditional media channels. This lack of differentiation appears to be a particular shortcoming of NHLFP consumer segments. Here, nontraditional media sources (e.g., social media platforms) function not only as a tool to gather information but also to enable users to engage in comparative social activities, which is an important prerequisite for NHLFP consumption. As a result, acquiring information becomes a “shared social experience” [72] that seems to drive media channel utilization rather than trust motives. 

While Cluster A members (“always skeptical”) do consider blueberries to be high priced (factor 10, item 3, μ = 0.79), which addresses the luxury price dimension of NHLFPs, they are unlikely to emphasize the luxury health dimension of NHLFPs due to their low degree of health awareness (μ = −0.49), lack of food knowledge, and low interest in comparing fruit consumption with their social network (factor 8, μ = −0.33). In comparison and as outlined previously, “foodies” have been found to regularly share food-related content due to their interest in and knowledge of food, thereby describing a prerequisite for the use of the health and experience dimensions of NHLFPs. Consequently, Cluster A shows no promising traits of consuming superfoods for luxury reasons and also has a low online marketing affinity and social media involvement; thus, it cannot be considered a promising consumer segment for future SMM strategies.

In comparison, Cluster B, the “media-skeptical light foodies”, displays high levels of regular fruit and blueberry consumption, which allows for a first conclusion, namely that a certain degree of familiarity with the price and possible health dimension of NHLFPs is present. This impression is strengthened due to high levels of health consciousness (factor 3, μ = 0.22) and members’ comprehensive knowledge of fruits (factor 11, item 1, μ = 0.98) and the health benefits of blueberries (factor 9, item 2, μ = 1.52). Yet, despite their awareness of the price and health dimension of NHLFPs, members are unlikely to utilize this for luxury reasons and, thus, to establish social class distinction. The reason is that their low desire to compare fruit consumption with their social network, in particular friends and family (factor 8, item 3, μ = −1.52; 4, μ = −1.30), suggests that they do not wish to be socially rewarded for their healthy food choice [28]. At the same time, while they regard blueberries as high priced, they strongly reject this as a luxury dimension (factor 10, μ = −0.12) that could be linked with the large share of high-income members within this cluster. This high income level differentiates members of Cluster B from Hartmann et al.’s [19] “sustainability- and health-oriented realists” consumer group who were also found to be health conscious but, at the same time, were not a promising target group for premium food marketing due to capital constraints. One must assume that the consumption pattern in Cluster B of NHLPFs is primarily driven by intrinsic motivational factors. An emerging field of research links personal well-being to healthy food consumption [73], which seems to be particularly relevant for fruit and vegetable products. Mujcucu and Oswald [74], Lesani et al. [75], and Blanchflower et al. [76] each find higher fruit and vegetable consumption, which blueberries are part of, to be linked to increased levels of personal happiness. While consuming for intrinsic health-related reasons, Cluster B members’ consumption motives might partly result from the circumstance that conducting healthy food choices by consuming blueberries also makes them happier. Overall, as Cluster B members revealed strong intrinsic consumption motives and no luxury preference concerning superfoods, they fit the “consumer” segment hypothesized by Sikka [28]. Simultaneously, they displayed similar characteristics to the “light-foodie” segment of Hemmerling et al. [29] due to their high knowledge of food topics and their concern that the media is not reporting frequently enough on these topics (factor 5, item 1).

Cluster C, the “traditionalists”, place high levels of trust in food-related information provided by the media, and this has apparently contributed to their high fruit (factor 11, μ = 0.38) and blueberry-related (factor 9, μ = 0.25) levels of knowledge compared to members of Cluster A who have low confidence in food-related media reporting as well as low levels of knowledge about fruits. However, “traditionalists” are unlikely to utilize NHLFPs for luxury reasons as they rarely use social media platforms that would support the communication of different NHLFP luxury dimensions, and they show no interest in consuming fruits for social comparison (factor 8, μ = −0.43). Nonetheless, members of Cluster C consume fruits and blueberries only slightly less often than members of Cluster B, despite reporting distinctly lower levels of health awareness (factor 3, μ = −0.12). Therefore, we assume that members consume fruits primarily as a result of their eating habits, as earlier literature on fruit consumption suggests [77]. This is likely to be the case as people in retirement represent the largest share of Cluster C members and elderly people are frequently advised to develop eating habits for health reasons [78]. Thus, while members of this cluster do not represent a promising target group for SMM and the advertising of NHLFP luxury dimensions, they might still represent an important consumer group for some superfoods.

In fourth place, members of Cluster D (“trustful luxury-seeking social foodies”) fit well with the “lifestyle” segment described by Sikka [28] and the “foodie” segment described by Hemmerling et al. [29], Johnston and Baumann [44], and Gunarathne et al. [45], as members have an above average level of health awareness and are fairly interested in and knowledgeable about food. In line with the previously identified foodie segments, members of Cluster D are also characterized by a larger share of females as well as a lower average age. Furthermore, members more often conduct food-related activities, such as fruit consumption, within their social network, which has previously been identified as a core characteristic of the foodie segment. Adding to our current knowledge on foodies, members of Cluster D place high importance on recognition from their social networks for healthy consumption patterns (factor 8, items 3 and 4). As hypothesized by Pampel et al. [13], this functions as a way to distinguish themselves from less health-conscious consumers and, thus, to establish social class differences by indicating that they belong to the particular social group previously identified by Sikka [28] as relevant for superfood consumption. Therefore, members are likely to link their high health consciousness and sensitivity toward environmentally friendly production practices with the described NHLFP dimension, in particular, credence dimensions, and use them as a luxury dimension. By doing so, members of Cluster D also differ from traditional luxury consumers who have in the past been found indifferent, almost skeptical, toward credence attributes, such as product sustainability, as a determinant of luxury consumption [19]. Furthermore, Cluster D has a strong affinity for social media and online marketing, which has been observed among foodies by other scholars [29,79]. Yet in comparison, social media usage among members of cluster D is much higher, likely fostering social comparisons among members even more strongly [29]. Pinterest, Instagram, and Facebook are more often used within Cluster D compared to previous foodie segments and the user average in Germany due to the strong association between these social media platforms and food topics or for comparing different lifestyle elements.

The phenomenon, which can been described as consumer citizen gap [80], is less likely to appear among members of Cluster D, as NHLFPs promote a larger degree of socially desirable behavior. The consumer citizen gap is likely to be small for premium food markets as prestige-seeking consumption motives outweigh price sensitivity. Members of Cluster D seem particularly exposed to this as they more often compare individual fruit consumption with their social networks, for example, family members (factor eight, item four, μ = 0.10), than do the other clusters, and they place the highest value on socially prestigious behavior, such as environmental awareness. Therefore, they are likely to pay a price premium in order to be recognized and socially rewarded for altruistic food consumption. Such behavior has also been observed with regard to other socially popular product groups such as organic food products. Seegebarth et al. [81] identified a consumer segment (“prestige seekers”) that strives to be recognized for socially desirable behaviors because they wish to be perceived as progressive consumers. Hartmann et al. [19] observed a similar desire among consumers of traditional luxury food products.

## 6. Conclusions, Limitations, and Further Research

As superfoods become more popular, they are increasingly used as luxury products. This has led to the emergence of a new product subcategory, namely NHLFP, which seems particularly prone to SMM. Due to its novelty, neither a coherent NHLFP definition nor corresponding consumer groups receptive to SMM strategies has yet been identified. For these reasons, this research raised two research questions. First, if the definition of NHLFP proposed at the beginning of this paper could be confirmed through empirical research. Second, whether or not there is a specific NHLFP consumer segment and if this segment is accessible through SMM strategies.

The proposed definition of NHLFP has set two conditions. First, associated food products must be of above average price and include an objective health benefit that can be identified through search and experience attributes. The case study of blueberries that was used fulfills these requirements. Research has linked blueberries to both the category of high-priced food products and superfoods [4], while they simultaneously display the necessary health-related search (e.g., blue color) and experience (e.g., bitterness in taste) attributes. Second, the definition of NHLFP presumes consumption for luxury reasons. While we were able to identify multiple consumer groups that consume blueberries regularly, members of Cluster D consume them for luxury reasons as well. Consequently, we can assume that the proposed definition—“New healthy luxury food products (NHLFPs) are traditional food products beneficial to a person’s health that are utilized as luxury products due to their above average product price and coexistence of health-related search and experience attributes”—is correct and provides a better understanding of consumption dynamics within the greater superfood category.

Out of the derived consumer groups, two clusters, the “media-skeptical light foodies” (24.68% of the data sample) and “trustful luxury-seeking social foodies” (16.36%) are identified as promising segments for superfood consumption, while the latter is also highly receptive to NHLFP consumption patterns. Furthermore, the “trustful luxury-seeking foodies” are found to make an ideal target group for SMM strategies as members display an explicit inclination toward socially comparable actions aided by high environmental awareness and high social media affinity. In the case of blueberries, members are highly receptive of search as well as credence product attributes and show tendencies to perceive these as luxury dimensions. Concerning the former, Cluster B members show no receptiveness to luxury food consumption as a means of displaying social class distinction. While they do consume blueberries, aided by distinct health and high environmental awareness, this happens for intrinsic health-promoting reasons.

Putting these results into practice means that members of Cluster B are not a promising target group for marketing strategies that want to promote NHFLPs through SMM. Their lack of luxury receptiveness and their focus on traditional sources of media advertisement implies to food business managers that price leadership strategies through traditional media sources, such as brochures, might be successful for targeting this consumer group. The “trustful luxury-seeking foodies” (Cluster D), on the other hand, make for an ideal target group. To ensure that marketing strategies are well tailored to this consumer group, marketing content needs to highlight luxury and the health-related search and credence attributes of NHLFP. Additionally, marketing strategies should build on novel marketing channels such as SMM. Ongoing campaigns by interest groups who market superfoods are right to increasingly build on social media platforms and to highlight superfood health benefits [35,39]. Public agencies should also consider this when designing future food education campaigns. Nevertheless, and this applies to all superfood producers and marketers who want to develop their products into NHLFP, a higher emphasis has to be put on accentuating the relationship between the objective health benefit of a product and other health-related product attributes (e.g., color, taste). For food business managers, this offers the opportunity to gain a specific competitive advantage if they are able to link, from the consumers point of view, the health benefits of their products well with their extrinsic and experience attributes. To exploit this, and in contrast to marketing strategies that address members of Cluster B, managers should execute a differentiation strategy when targeting Cluster D affiliated consumers [82]. Marketing strategies also need to enable consumers to more easily compare and share their NHLFP eating habits, especially across social media platforms. Nowadays, consumers require more engaging types of marketing, and the NHLFP target group is likely to be at the frontier of this development. A possible marketing direction that is more engaging and builds on social media platforms could be the provision of cooking recipes through short video clips as well as reliance on influencer marketing because influencers provide highly trusted [83,84,85] and engaging marketing content. Furthermore, the ongoing coronavirus pandemic has led to higher awareness among consumers regarding the sustainability of product packaging designs and storage processes [17,18]. Therefore, marketing strategies that focus on sustainable NHLFP packaging and the transforming of designs as a luxury criterion is a good way forward. Corresponding advertisement could either teach consumers about how to store products in a sustainable way or highlight equivalent characteristics that are already part of the product. Both would provide engaging marketing content and allow consumers to easily compare these activities with others.

As is the case with most exploratory studies, possible shortcomings of this research have to be addressed. First, data collection was conducted outside the traditional blueberry season within Germany, and this might have impeded people’s knowledge of blueberries and surely their indicated consumption frequency. In fact, data collection took place during a time of high social restrictions in Germany, which we know has influenced food consumption patterns and likely social media usage [17,18]. Second, the data are not entirely representative for all income and educational subgroups, which can possibly bias results as both characteristics influence health awareness and food consumption patterns and, thus, NHLFP attitudes. Data collection was carried out with the assistance of an online panel provider, which is oftentimes helpful because it speeds up the actual data collection but allows no conclusion on the response rate and, therefore, the possible interest in this topic. From a statistical point of view, cluster analysis can lead to spurious observations of similarities within a given data set. This could be reinforced by the relatively small sample size of this study (see Section 4.1) [54,55]. We are confident that this is not the case in our study according to the recommended quality tests (see Section 4.3) [86]. Generally, the number of identified clusters can vary depending on the methodological criteria chosen [86]. By analyzing dendrogram and scree plot outputs next to the WARD criteria (see Section 3.2), we minimized the risk of biased results. 

In terms of future research, NHLFP consumption patterns should be investigated with new, increasingly interdisciplinary research methods in food marketing [73,87]. As intrinsic happiness-driven motives might influence NHLFP consumption, neuromarketing approaches in food choices allow researchers to measure corresponding behavior such as emotional consumer response directly [87] to enhance the understanding of NHLFP consumption patterns. Extended research is also needed on the proposed concept of NHLFP and should try to identify other superfoods which fit this product subcategory. While this research focused on a high-income society by using Germany as an example, future work could investigate if similar observations can be made for middle-income countries. Future research could also investigate similarities among NHLFP consumers more strongly for which correspondence analysis could be applied. Additionally, different social media platforms, such as bookmarking websites and relationship networks, have significantly shaped cluster affiliation in this study, and it would be interesting to investigate in the future the differences in their potential to influence NHLFP perceptions. With this in mind, we have seen that our current work horses for measuring media trust and linking it with the use of different media sources fall short in explaining today’s user dynamics. Future research needs to provide a more nuanced picture by observing trust-based media differences within individual modern media channels.

## Figures and Tables

**Table 1:** Descriptive sociodemographic representation of the data sample.

	Sample ^1^	German Population ^2^
**Gender (%)**	Female: 51.2 Male: 48.8	Female: 50.7 Male: 49.3
**Age Ø (%)**		
**18–24 years**	9.2	7.6
**25–34 years**	15.8	12.8
**35–44 years**	15.6	12.4
**45–59 years**	22.1	22.7
**60 years and older**	37.3	28.7
**Place of living (%) ^3^**	North: 18.9 East: 19.5 West: 35.4 South: 26.1	North: 18.1 East: 17.6 West: 35.3 South: 28.9
**Household income after taxes (%)**		
	Under €900: 18.5	Less than €900: 7.9
	€900 to 1499: 19.5	€901–1500: 16.5
	€1500 to 1999: 17.9	€1501–2000: 14.9
	€2000 to 2499: 16.9	€2001–2600: 15.7
	€2500 to 2999: 9.9	€2600–3200: 11.6
	€3000 to 3499: 7.6	€3200 and more: 33.4
	€3500 and more: 9.6	
**Education ^4^**		
Brief	18.2	35.0
Middle	36.3	31.1
High	45.5	33.88
		
**Health** [%]		
Smoking cigarettes	19.2	17.53
Sport activity	57.2	56.9 ^5^
Alcohol consumption	33.3	69 ^6^
		
**Fruit consumption** [%]		
Daily	39.4	31.00 ^7^
Multiple times per week	49.3	45.41 ^7^
Once per week	11.3	10.27 ^7^
**Dietary preference**		
Vegetarian	8.2	6.5
Vegan	3.4	1.13
		
**Internet affinity ^8^**		
Ø minutes online per day	191.28	196
Ø minutes on social media per day	41.5	79

^1^ Data sample (*N* = 697). ^2^ Values based on [52,56,57,58,59,60,61,62,63,64,65]. ^3^ North: Schleswig-Holstein, Hamburg, Bremen, Lower Saxony, Mecklenburg-Vorpommern; East: Berlin, Brandenburg, Saxony-Anhalt, Saxony, Thuringia; South: Bavaria, Baden-Wuertemberg; West: North Rhine-Westphalia, Hessen, Rhineland-Palatinate, Saarland. ^4^ Brief education: no school leaving certificate/lower secondary school/primary school; Middle education: secondary school, polytechnic school, master school; Higher education: grammar school, university (the highest achieved level of education had to be indicated). ^5^ Includes at least once per week. ^6^ Includes consumption of up to once per week. ^7^ German population from 14 years and older. ^8^ Corrected for unrealistic outliers. Source: Authors’ calculation. Bold indicates categories which are followed by different subcategories.

**Table 2:** Exploratory factor analysis ^1^.

Factor 1: Media Quality Perception I (Cronbach’s Alpha: 0.910; Explained Share of Variance: 14.05%) ^2^	Factor Loading	μ	σ
1.1. In the German media landscape, journalists’ opinions are well founded	0.888	0.09	0.898
1.2. In the German media landscape, journalists express criticism in an adequate manner	0.821	0.06	0.881
1.3. When talking about food, the media provide all important information regarding current topics	0.819	0.13	0.943
1.4. When talking about food, the media reporting included different points of view	0.815	0.09	0.908
1.5. When talking about food, the media coverage’s focus is on important facts	0.795	0.28	0.914
1.6. When talking about food, the media address the essential points of the topics	0.642	0.28	0.895
**Factor 2: Environmental Awareness (Cronbach’s Alpha: 0.897; Explained Share of Variance: 20.41%) ^2,3^**	**Factor Loading**	**μ**	**σ**
2.1. When there is a choice, I choose the product that causes the least pollution	0.870	0.84	1.003
2.2. I avoid buying products that have excessive packaging	0.851	0.94	1.003
2.3. I make every effort to buy paper products made from recycled paper	0.812	0.58	1.049
2.4. It is important to me that food products are grown in an environmentally friendly way	0.782	0.73	0.958
2.5. Whenever possible, I buy products packaged in reusable containers	0.774	0.73	0.963
2.6. I use environmentally friendly soaps and detergents	0.720	0.28	1.150
2.7. I pay attention to how food is produced before I purchase it	0.654	0.16	1.017
**Factor 3: Health Awareness (Cronbach’s Alpha: 0.889; Explained Share of Variance: 8.3%) ^2^**	**Factor Loading**	**μ**	**σ**
3.1. I’m aware of the state of my health as I go through the day	0.873	0.34	1.042
3.2. I notice how I feel physically as I go through the day	0.841	0.57	1.033
3.3. I’m very involved with my health	0.818	0.05	1.139
3.4. I’m constantly examining my health	0.812	0.38	1.082
3.5. I’m alert to changes in my health	0.750	0.56	0.951
3.6. I’m generally attentive to my inner feeling about my health	0.731	0.90	0.878
**Factor 4: Social Media Involvement (Cronbach’s Alpha: 0.892; Explained Share of Variance: 5.74%)**	**Factor Loading**	**μ**	**σ**
4.1. I often use social media to inform myself about upcoming events	0.916	−0.50	1.352
4.2. I frequently use social media to inform myself about events that have taken place	0.818	−0.79	1.224
4.3. Social media helps me a lot with improving my knowledge about companies that interest me	0.763	−0.75	1.256
4.4. Friends use social media to contact me	0.753	0.09	1.433
4.5. I often use social media to inform myself about products that interest me	0.709	−0.76	1.287
**Factor 5: Media Quality Perception II (Cronbach’s Alpha: 0.920; Explained Share of Variance: 4.78%) ^2^**	**Factor Loading**	**μ**	**σ**
5.1. When regarding food, the German media report on food topics in an adequate frequency	0.871	0.19	0.949
5.2. When regarding food, the German media report on important topics on the necessary regular basis	0.868	0.34	0.917
5.3. When regarding food, the German media pay the necessary attention to important topics	0.846	0.19	0.939
5.4. When regarding food, the German media assign important topics an adequate status	0.841	0.17	0.942
**Factor 6: Extrinsic Blueberry Characteristics (Cronbach’s Alpha: 0.742; Explained Share of Variance: 4.16%)**	**Factor Loading**	**μ**	**σ**
6.1. The cultivation of blueberries is particularly environmentally friendly	0.784	−0.07	0.749
6.2. The cultivation of blueberries requires the application of a small amount of pesticides	0.774	−0.09	0.789
6.3. Compared to other fruits, blueberries are particularly regional	0.726	−0.26	0.970
6.4. Blueberries have seals of quality that are known to me	0.633	−0.33	0.945
6.5. On the packaging of blueberries, meaningful product information can be found	0.595	0.05	0.867
**Factor 7: Online Marketing Affinity (Cronbach’s Alpha: 0.791; Explained Share of Variance: 3.26%) ^3^**	**Factor Loading**	**μ**	**σ**
7.1. I often purchase products that were shown to me through advertising on the Internet before	0.900	−1.33	0.924
7.2. I regularly click on advertisements that are displayed to me on the Internet	0.818	−1.28	0.971
7.3. Advertising that is displayed on the Internet to me frequently addresses food products	0.784	−0.90	0.982
**Factor 8: Social Comparison of Fruit Consumption (Cronbach’s Alpha: 0.767; Explained Share of Variance: 2.92%)**	**Factor Loading**	**μ**	**σ**
8.1. Oftentimes, I eat fresh fruit directly before or after doing sports	0.778	−0.39	1.254
8.2. I eat fresh fruits when I’m with friends	0.755	−0.34	1.098
8.3. Sometimes I consume fresh fruit to show my friends how health conscious I live	0.732	−1.34	0.982
8.4. Sometimes I consume fresh fruit to show my family how health conscious I live	0.665	−1.20	1.067
**Factor 9: Intrinsic Blueberry Characteristics (Cronbach’s Alpha: 0.687; Explained Share of Variance: 2.52%)**	**Factor Loading**	**μ**	**σ**
9.1. Fresh blueberries taste aromatically sweet to slightly sour	0.837	1.24	0.721
9.2. Fresh blueberries contain a high amount of healthy nutritive substances	0.737	1.29	0.706
9.3. Fresh blueberries have an intensive blue coloration	0.683	1.04	0.765
9.4. Fresh blueberries smell sweetish	0.615	0.49	0.860
**Factor 10: Luxury Perception of Blueberries (Cronbach’s Alpha: 0.641; Explained Share of Variance: 2.46%)**	**Factor Loading**	**μ**	**σ**
10.1. Compared to other fresh fruit, blueberries are often only consumed on special occasions	0.818	−0.02	1.143
10.2. Compared to other fresh fruit, blueberries are particularly exclusive	0.736	0.29	1.000
10.3. Blueberries are high prices compared to other fresh fruit	0.714	1.00	0.298
**Factor 11: Fruit Knowledge (Cronbach’s Alpha: 0.565; Explained Share of Variance: 2.33%)**	**Factor Loading**	**μ**	**σ**
11.1. I’m well aware of the different fruits available when shopping in my local groceries stores	0.823	0.80	0.201
11.2. I’m well acquainted with the product characteristics of the fresh fruit I buy	0.747	0.33	0.978
11.3. I know more about other foods that I do about fresh fruit ^3^	0.594	0.19	0.876

^1^ All variables were sampled using 5-point Likert scales (from +2 = fully agree to −2 = fully disagree) and were evaluated using principal component factor analysis with ProMax rotation allowing for 10 iterations. Kaiser–Meyer–Olkin (KMO) value = 0.880, Bartlett significance level: 0.000; total explained share of variance: 65.64%. ^2^ Items were carefully translated into German with the assistance of a native English speaker. ^3^ Reverse recoded variable. Source: Authors’ calculation.

**Table 3:** Hierarchical cluster analysis ^1^.

	Cluster A *n* = 205 (29.41%)	Cluster B *n* = 172 (24.68%)	Cluster C *n* = 206 (29.56%)	Cluster D *n* = 114 16.36%)
	μ	μ	Μ	μ
Factor 1: Media Quality Perception I ***	−0.28 ^b,c,d^	−0.75 ^a,c,d^	0.59 ^a,b^	0.56 ^a,b^
Factor 2: Environmental Awareness ***	−0.78 ^b,c,d^	0.49 ^a,c^	0.05 ^a,b,d^	0.57 ^a,c^
Factor 3: Health Awareness ***	−0.49 ^b,c,d^	0.22 ^a,c,d^	−0.12 ^a,b,d^	0.78 ^a,b,c^
Factor 4: Social Media Involvement ***	−0.12 ^c,d^	0.00 ^c,d^	−0.41 ^a,b,d^	0.92 ^a,b,c^
Factor 5: Media Quality Perception II***	−0.27 ^b,c,d^	−0.96 ^a,c,d^	0.82 ^a,b,d^	0.45 ^a,b,c^
Factor 6: Extrinsic Blueberry Characteristics ***	−0.44 ^b,c,d^	−0.06 ^a,d^	−0.16 ^a,d^	1.16 ^a,b,c^
Factor 7: Online Marketing Affinity ***	−0.02 ^a,b,c^	−0.37 ^a,d^	−0.37 ^a,d^	1.27 ^a,b,c^
Factor 8: Social Comparison of Fruit Consumption ***	−0.33 ^b,d^	0.05 ^a,c,d^	−0.43 ^b,d^	1.29 ^a,b,c^
Factor 9: Intrinsic Blueberry Characteristics ***	−0.64 ^b,c,d^	0.21 ^a^	0.25 ^a^	0.39
Factor 10: Luxury Perception of Blueberries ***	−0.05 ^d^	−0.12 ^d^	−0.17 ^d^	0.57 ^a,b,c^
Factor 11: Fruit Knowledge ***	−0.83 ^a,b,c^	0.39 ^a^	0.38 ^a^	0.22 ^a^

^1^ Level of significance: *** = *p* ≤ 0.01; Letters (a,b,c,d) represent a significant difference to the corresponding cluster (Tamhane post-hoc multiple comparison test at significant level 0.05). All variables were sampled using a 5-point Likert-scale (from +2 = fully agree to −2 = fully disagree). Source: Authors’ calculation.

**Table 4:** Cluster description: sociodemographic, lifestyle and food consumption patterns across clusters.

	Cluster A *n* = 205 (29.41%)	Cluster B *n* = 172 (24.68%)	Cluster C *n* = 206 (29.56%)	Cluster D *n* = 114 (16.36%)	Pearson Chi-Quadrat	Asymp. Sig. (Bilateral)
Sociodemographic characteristics						
Age Ø in years ^1^	47.28	48.34	56.85	43.1	300.059	0.000
Share of woman (%) ^1^	42.9 **	56.4	53.4	54.4	8.335	0.040
						
**Place of living** (%) ^1,2^					53.439	0.182
North	16.0	16.9	22.3	21.1		
East	28.3 **	16.2	17.1	13.3		
West	33.7	33.7	34.9	42.1		
South	22.0	33.1	25.7	23.5		
**Household income after taxes** (%) ^1,3^					20.877	0.286
Under €900	8.8	8.7	6.7	4.0		
€900 to €1499	13.5	9.9	11.8	13.9		
€1500 to €1999	13.0	13.0	8.7	15.7		
€2000 to €2499	15.0	12.4	16.9	21.8		
€2500 to €2999	9.8	14.9	19.0	14.9		
€3000 to €3499	14.5	11.2	13.8	8.9		
€3500 and more	25.4	29.8	23.1	20.8		
**Education** (%) ^1,4^					29.831	0.095
Brief education	17.1	19.8	16.4	21		
Middle education	40.0	33.1	39.9	28		
Higher education	42.9	47.1	43.7	50.9		
**Occupation** (%) ^1^					90.418	0.000
Student/Trainee	5.8	14.0	5.8	17.5 *		
Employee	54.1 ***	34.8	26.7 ***	47.4		
Self-employed	3.9	4.7	10.2 ***	0.0		
Public service	13.7	15.1	14.1	10.6		
Housewife/men	1.0	8.7 ***	3.4	3.5		
Retired	21.5	22.7	39.8	21.0		
Healthy lifestyle (%)						
Smoke	17.1	17.4	20.9	22.8	2.266	0.519
Exercise regularly	47.3 ***	69.8 ***	49.5 *	70.2 **	32.656	0.000
Consume alcohol regularly	32.2	27.9	37.4	36.0	4.273	0.233
Food consumption patterns						
Familiarity with blueberry characteristics (%) ^5^	14.7 ***	41.9	47.5 **	64.00 ***	115.174	0.000
**Dietary preference** (%) ^1^						
Vegan	1.5	8.7 ***	0.5 *	4.4	37.869	0.000
Vegetarian	3.8	7.6	12.6 *	8.8		
Meat eater	85.9	72.1 *	81.1	76.3		
Other	8.8	11.6	5.8	10.5		
**Blueberry consumption** (%) ^1^					15.743	0.610
Daily	0.9	0.00	1.9	3.5		
Multiple times per week	14.2	12.2	11.2	14.9		
Once per week	13.2	16.3	12.1	15.8		
Repeatedly within one month (but not every week)	18.5	19.2	17.9	18.4		
Approximately once a month	12.7	13.4	9,7	7.0		
Less than once a moth	33.7	31.9	39.3	30.7		
Never	6.8	6.9	7.7	9.7		
**Fruit consumption** (%) ^1^						
Daily	36.6	45.4	38.4	36.8	9.066	0.170
Multiple times per week (but not daily)	49.3	44.8	49.0	57.0		
Once per week	14.2	9.9	12.6	6.1		
Repeatedly within one month (but not every week)	0.00	0.00	0.00	0.00		
Approximately once a month	0.00	0.00	0.00	0.00		
Less than once a moth	0.00	0.00	0.00	0.00		
**Fruit purchase frequency** (%) ^1^					18.935	0.396
Daily	3.4	1.7	1.9	6.1		
Multiple times per week	40.9	40.7	34.5	45.6		
Once per week	43.9	47.1	53.8	40.4		
Repeatedly within one month (but not every week)	7.3	6.9	7.8	5.3		
Approximately once a month	1.9	2.3	1.9	1.8		
Less than once a moth	2.5	0.0	0.0	0.9		
**Place of fruit purchase** (%) ^1^					40.513	0.046
Discounter	38.0	23.8	25.7	23.7		
Supermarket/convenience store	52.7	52.3	58.7	57.9		
Farmers market/Sustainable production focused stores	8.8	22.7	15.1	18.5		
Internet	0.0	0.6	0.5	0.0		
Other	0.5	0.6	0.0	0.0		

Level of significance: * = *p* ≤ 0.1, ** = *p* ≤ 0.05, and *** = *p* ≤ 0.01 indicate a significant difference between clusters between the expected and observed quantity. For all items, the Bonferroni adjustment has been applied to prevent type I errors. ^1^ Only one answer was possible regarding the represented question. ^2^ North: Schleswig-Holstein, Hamburg, Bremen, Lower Saxony, Mecklenburg-Vorpommern; East: Berlin, Brandenburg, Saxony-Anhalt, Saxony, Thuringia; South: Bavaria, Baden-Wuertemberg; West: North Rhine-Westphalia, Hessen, Rhineland-Palatinate, Saarland. ^3^ Independent of the marital status and resulting possible adjustments of the household income structure. ^4^ Brief education: no school leaving certificate/lower secondary school/primary school; Middle education: secondary school, polytechnic school, master school; Higher education: grammar school, university (the highest achieved level of education had to be indicated). ^5^ Question “I’m very familiar with the characteristics of fresh blueberries” queried on a 5-point Likert scale (from +2 = fully agree to −2 = fully disagree). Characteristics 2 = fully agree and 1 = agree were aggregated and are displayed here. Source: Authors’ calculation. Bold is to distinguishing categories from subcategories.

**Table 5:** Cluster description: digital and social media affinity across clusters.

	Cluster A *n* = 205 (29.41%)	Cluster B *n* = 172 (24.68%)	Cluster C *n* = 206 (29.56%)	Cluster D *n* = 114 (16.36%)	Pearson Chi-Quadrat	Asymp. Sig. (Bilateral)
Digital and social media affinity						
**Digital hardware (%)**						
Smartphone	91.2	94.7 *	86.4	88.6	8.014	0.046
Tablet	53.2	46.5	50.0	49.2	1.695	0.638
Laptop	76.6	79.1	71.4	80.7	4.745	0.191
Installed Computer	50.7	52.9	52.9	50.9	0.314	0.957
Television	76.1	73.8	83.1 *	73.7	5.995	0.112
**Intensity of Internet usage** ^1^					213.266	0.263
Using the Internet on average more than 60 min each day (%)	75.1	71.5	78.5	71.9		
Using the Internet on average more than 200 min each day (%)	35.1	36.6	30.1	32.5		
Mean average of minutes spend on social media per day	42.3	44.5	28.3	58.5		
Usage of different media platforms (%) ^2^ (using this media for price information of (%) ^3^/health information (%) ^4^ on blueberries)						
Daily and weekly newspaper	48.8 *** (4.4/6.4)	57.6 (3.5/4.6)	70.4 *** (13.1/16.5)	64.9 (24.6/27.2)	21.583 78.090/57.670)	0.000 (0.000/0.000)
Advertising brochures	69.8 (60.5/5.9)	59.9 ** (55.8/7.0)	72.8 (65.0/9.7)	71.9 (67.6/28.9)	8.477 (36.743/88.855)	0.037 (0.000/0.000)
Television and streaming platforms	75.6 (3.4/12.2)	72.1 (1.7/12.2)	76.2 (3.4/17.9)	85.1 * (24.6/43.8)	6.628 (138.253/88.236)	0.085 (0.000/0.000)
Radio	76.6 (1.0/3.0)	79.7 (1.8/4.7)	85.0 (2.5/7.8)	81.6 (27.2/22.8)	4.782 (148.860/72.790)	0.188 (0.000/0.000)
Professional journals	25.4 *** (2.0/24.3)	41.3 (2.9/33.7)	39.8 (1.5/25.7)	40.4 (17.5/40.3)	14.372 (95.173/42.611)	0.002 (0.000/0.000)
Relationship networks (Facebook, LinkedIn, Xing)	57.1 (0.5/2.5)	56.4 (1.2/6.4)	46.1 *** (0.5/1.5)	75.4 *** (14.1/14.0)	25.717 (133.513/121.808)	0.000 (0.000/0.000)
Platforms for sharing pictures (Instagram, Flickr)	32.2 (1.5/1.5)	36.6 (0.6/2.3)	20.4 *** (0.0/0.00)	51.8 *** (13.2/14.9)	34.039 (148.620/118.301)	0.000 (0.000/0.000)
Platforms for sharing videos (YouTube, SnapChat)	59.0 (2.9/4.9)	59.9 (1.2/8.7)	52.9 * (0.5/2.9)	71.1 ** (17.5/20.1)	10.042 (132.172/92.027)	0.018 (0.000/0.000)
Blogs	18.0 (2.9/4.4)	22.7 (3.5/9.8)	19.4 (1.0/4.4)	36.8 *** (12.3/21.1)	16.802 (87.739/79.294)	0.001 (0.000/0.000)
Microblogging (Twitter, Vine)	12.2 (1.5/1.5)	11.6 (1.7/2.9)	9.2 (1.0/0.5)	16.7 (6.1/10.5)	3.905 (101.121/96.997)	0.272 (0.000/0.000)
Bookmarking websites (Pinterest)	15.6 (1.5/3.0)	16.3 (1.2/4.7)	13.1 (0.5/1.0)	32.5 *** (11.4/11.4)	20.790 (115.472/89.363)	0.000 (0.000/0.000)
Interest-based networks	6.8 (1.0/3.9)	8.7 (1.2/7.6)	9.7 (0.5/6.3)	19.3 *** (11.4/18.4)	13.326 (97.042/83.385)	0.004 (0.000/0.000)
Recommendation portals (Yelp, TripAdvisor)	23.9 (2.0/2.0)	25.0 (1.7/1.8)	26.2 (0.5/2.4)	35.1 (8.8/10.5)	5.198 (83.237/77.233)	0.158 (0.000/0.000)

Level of significance: * = *p* ≤ 0.1, ** = *p* ≤ 0.05, *** = *p* ≤ 0.01 indicate a significant difference between clusters between the expected and observed quantity. For all items, the Bonferroni adjustment has been applied to prevent type I errors. ^1^ Only one answer was possible regarding the represented question. ^2^ First, participants were asked to indicate the different media types that they regularly use. ^3^ The question “If I would like to inform myself about the current sales price of blueberries, I would choose [different media types]” queries on a 5-point Likert scale (from +2 = fully agree to −2 = fully disagree). Characteristics 2 = fully agree and 1 = agree were aggregated and are displayed here. ^4^ The question “If I would like to inform myself about the health benefits of blueberries, I would choose … [different media types]” queries on a 5-point Likert scale (from +2 = fully agree to −2 = fully disagree). Characteristics 2 = fully agree and 1 = agree were aggregated and are displayed here. Source: Authors’ calculation.

## Data Availability

The utilized questionnaire and data presented in this study are available upon reasonable request. Please contact the corresponding author.
